# Ketamine Impact on Kidney Health

**DOI:** 10.7759/cureus.70804

**Published:** 2024-10-04

**Authors:** Sana Rahman, Samiya Saher, Anurag Raje, Suriya Shanmugar, Isha Gupta

**Affiliations:** 1 Internal Medicine, Osmania Medical College, Hyderabad, IND; 2 Internal Medicine, Narendra Kumar Prasadrao (NKP) Salve Institute of Medical Sciences, Nagpur, IND; 3 Internal Medicine, ACS Medical College and Hospital, Chennai, IND; 4 Nephrology, Middletown Medical, Middletown, USA; 5 Nephrology, Garnet Health Medical Center, Middletown, USA; 6 Internal Medicine/Nephrology, Touro College of Osteopathic Medicine, Middletown, USA

**Keywords:** bilateral hydronephrosis, burning micturition, ckd (chronic kidney disease), dysuria, flank pain, hydroureteronephrosis, ketamine-associated cystitis, ketamine-induced uropathy, renal failure, ureteral stent

## Abstract

Ketamine-induced uropathy (KIU) is a serious consequence of chronic ketamine abuse, presenting with complex renal and urinary symptoms. This study describes a 34-year-old female with a history of chronic ketamine abuse, resulting in stage 3 chronic kidney disease (CKD) and severe urological complications. Despite discontinuing ketamine use five years ago, she remains dependent on ureteral stents due to recurrent hydronephrosis and ureteral obstruction. The patient began using ketamine at the age of 25 years, consuming approximately 5 g daily for two years. By the age of 27 years, she developed dysuria, flank pain, and burning micturition and was later diagnosed with ketamine-associated cystitis and renal failure secondary to hydronephrosis. Initially, bilateral ureteral stents were placed to manage her condition, but she continued to experience worsening symptoms. Although studies suggest that early cessation of ketamine can resolve ulcerative cystitis and ureteral obstruction, this was not observed in our patient.

This case highlights the importance of high suspicion for ketamine abuse in young patients presenting with ureteral complications such as hydronephrosis and cystitis-like symptoms. It highlights the need for early detection, ongoing follow-up, and a comprehensive approach involving pharmacological and surgical interventions. Effective management also requires counseling on ketamine discontinuation to prevent further and permanent damage to the urinary system.

## Introduction

Ketamine is a general anesthetic; at subanesthetic doses, it produces a dissociative state characterized by a sense of detachment from one's physical body and the external world, known as depersonalization and derealization [1]. Ketamine is a non-competitive N-methyl-D-aspartate antagonist metabolized in the liver to nor ketamine. This active metabolite of ketamine is then excreted renally [2]. It is commonly abused as a recreational drug at clubs and parties. There is documented evidence suggesting large amounts of ketamine cause urinary toxicity and liver toxicity. Ketamine, which was initially developed as a safer alternative to phencyclidine, is used in clinical settings for analgesia and sedation [3]. In recent years, its applications have expanded to include pain management and treatment for asthma and depression. Chronic ketamine abuse is associated with a condition known as ketamine-associated cystitis or ketamine-induced ulcerative cystitis, which can extend its impact on the ureters and kidneys. It can cause inflammation and ulceration of the bladder wall, which may extend to the ureters. Ketamine-associated lower-urinary tract symptoms include frequency, urgency, nocturia, dysuria, urge incontinence, and occasionally painful hematuria [4].

## Case presentation

A 34-year-old female with a history of chronic ketamine abuse, which began at the age of 25 years with daily consumption of approximately 5 g for two years, presented at the age of 27 years with worsening bilateral flank pain, severe back pain, dysuria, general malaise, and burning micturition. Laboratory tests at that time revealed a creatinine level of 6.9 mg/dL and a potassium level of 5.3 mEq/L, prompting the recommendation for dialysis, which she declined. During this time, the patient reported significantly elevated liver function tests (LFTs) with levels purportedly in the thousands, though no records were available for verification. A liver biopsy was advised, but the patient refused.

Subsequently, the patient sought another urologist due to persistent pressure with urination and urgency issues. During this visit, her creatinine was 4 mg/dL, and her past history of recreational ketamine use was identified as a causative factor for her ureteral blockage, which was confirmed on cystoscopy showing erythematous congestion and ulceration, and a biopsy showing urothelial denudation, edema, and mast cells. Based on these findings, she was diagnosed with ketamine-associated cystitis, complicated by renal failure secondary to obstructive hydronephrosis. Bilateral ureteral stents were placed to relieve the ureteral obstruction causing hydronephrosis and the creatinine levels decreased to 1.27 mg/dL. However, she did not attend follow-up appointments, leaving the stents indwelling.

Three years later, she presented with worsening flank pain, burning, and pain during micturition. Her creatinine level had increased to 1.4 mg/dL, raising concerns that the stents were malfunctioning. Their removal was expected to resolve her symptoms, so the stents were removed.

**Figure 1:**
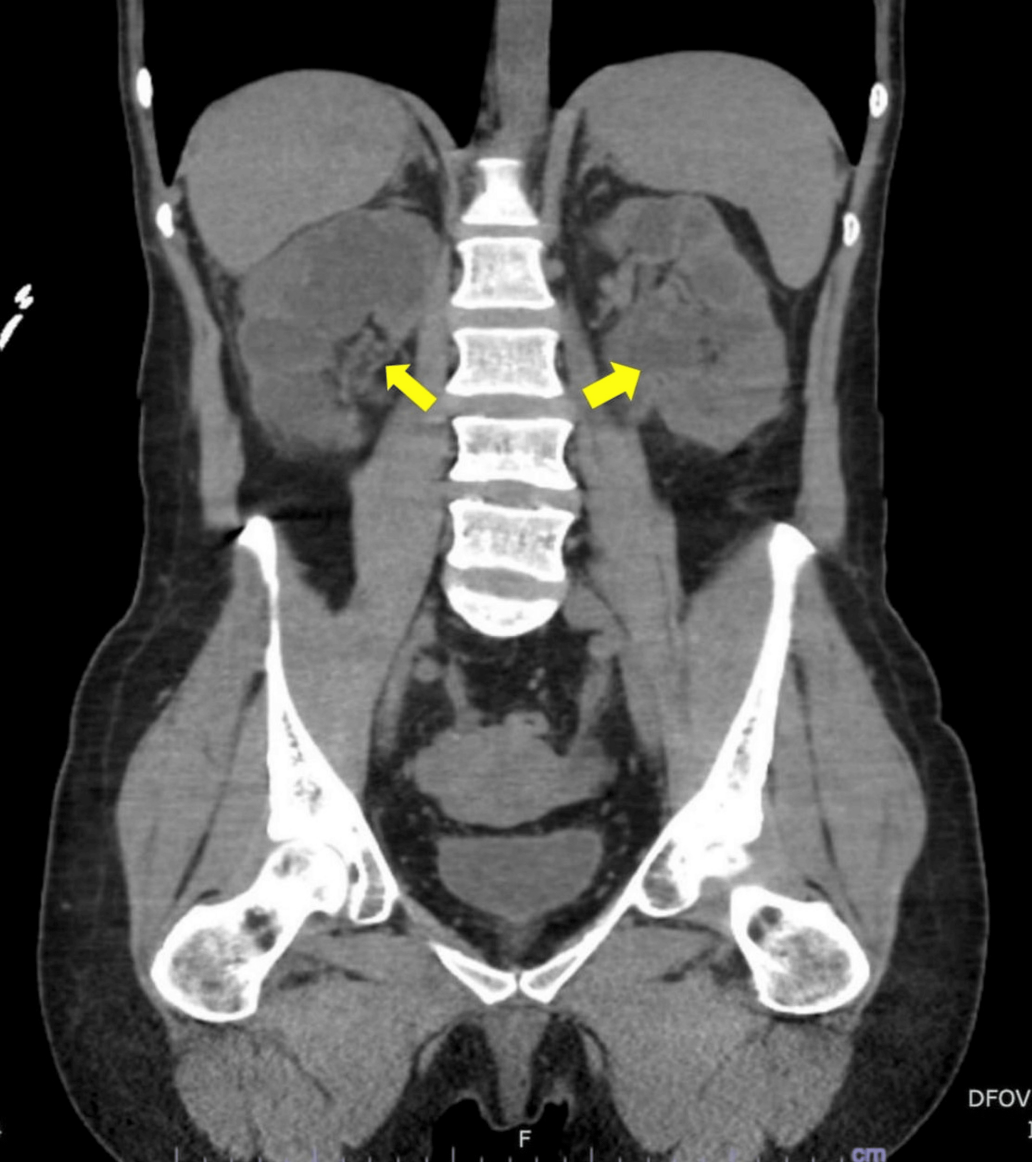
A coronal view of CT abdomen pelvis without IV contrast showing hydronephrosis before stenting.

**Figure 2:**
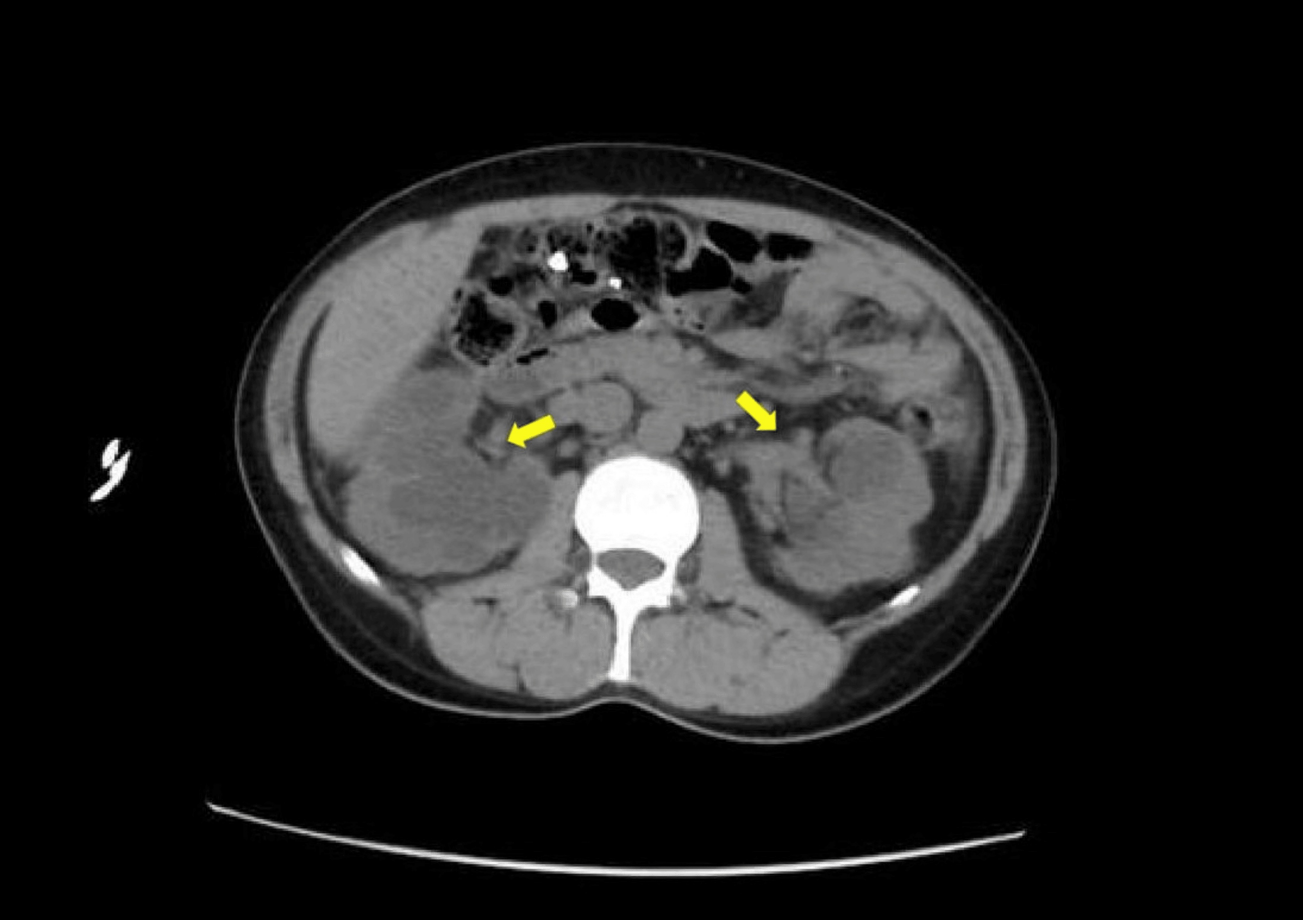
A cross-sectional view of CT abdomen pelvis without IV contrast showing hydronephrosis before stenting.

Despite stent removal, her condition worsened three days later, leading to an emergency room visit with severe flank pain, nausea, and hematuria. A CT scan of the abdomen and pelvis showed bilateral hydroureteronephrosis, perinephric and periureteral fat stranding, and renal cortical thinning (Figures 1, 2). Laboratory results revealed sepsis with worsening hydronephrosis, with creatinine and estimated glomerular filtration rate (eGFR) levels at 2.27 mg/dL and 28.3 mL/min/1.73 m², respectively, indicating worsening renal function (Table 1). She was also seen by an infectious disease specialist due to a complicated UTI, evidenced by fever and leukocytosis, and was treated with IV antibiotics, later transitioning to oral antibiotics. Blood and urine cultures were negative. Her creatinine peaked at 2.36 mg/dL during this admission.

**Table 1:** Lab values of the patient after removal of the indwelling non-functional stents and post re-stenting.

Laboratory tests	Pre-procedure (June 6, 2024)	Post-procedure (June 10, 2024)	Normal values
WBC	13.2 x 10^3^/µL	7.4 x 10^3^/µL	4-11 x 10^3^/µL
RBC	4.10 x 10^6^/µL	3.99 x 10^6^/µL	4.35-5.65 x 10^6^/µL
Hemoglobin	11.7 g/dL	11.1 g/dL	11.6-15 g/dL
Hematocrit	36.0%	35.1%	36-48%
Platelets	215 x 10^3^/µL	257 x 10^3^/µL	150-450 x 10^3^/µL
Sodium	136 mEq/L	138 mEq/L	135-145 mEq/L
Potassium	4.3 mEq/L	3.7 mEq/L	3.5-5 mEq/L
Chloride	108 mEq/L	108 mEq/L	95-105 mEq/L
CO_2_	17 mEq/L	22 mEq/L	22-30 mEq/L
BUN	16 mg/dL	9 mg/dL	6-24 mg/dL
Glucose	96 mg/dL	91 mg/dL	70-100 mg/dL
Calcium	8.4 mg/dL	8.3 mg/dL	8.5-10.5 mg/dL
Creatinine	2.27 mg/dL	1.49 mg/dL	0.6-1.2 mg/dL
Anion gap	11 mEq/L	8 mEq/L	6-12 mg/dL
eGFR female	28.3 mL/min/1.73 m^2^	47.0 mL/min/1.73 m^2^	>90 mL/min/1.73 m^2^

**Figure 3:**
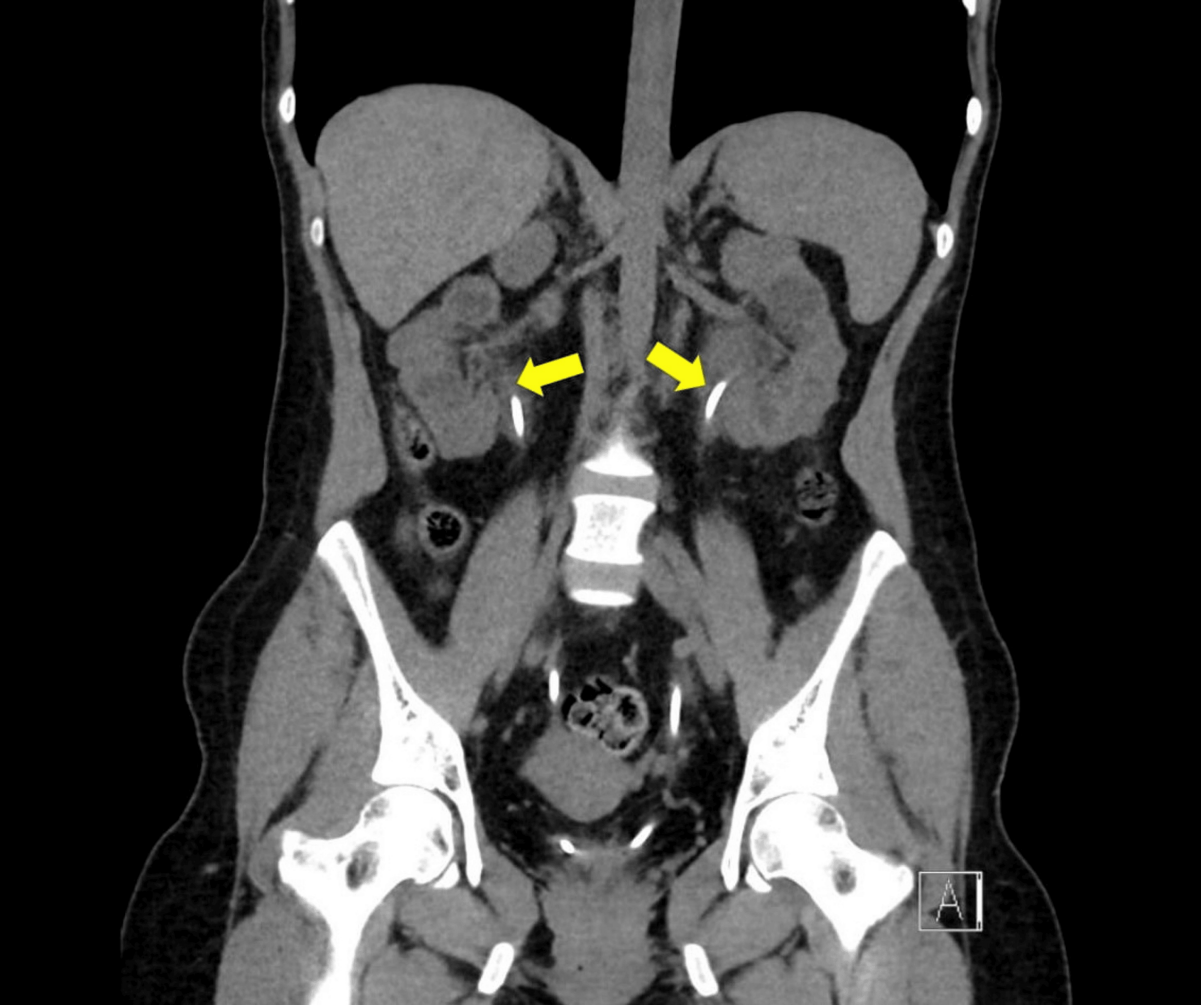
A coronal view of CT abdomen and pelvis without IV contrast showing the resolution of obstruction after placement of ureteral stents.

**Figure 4:**
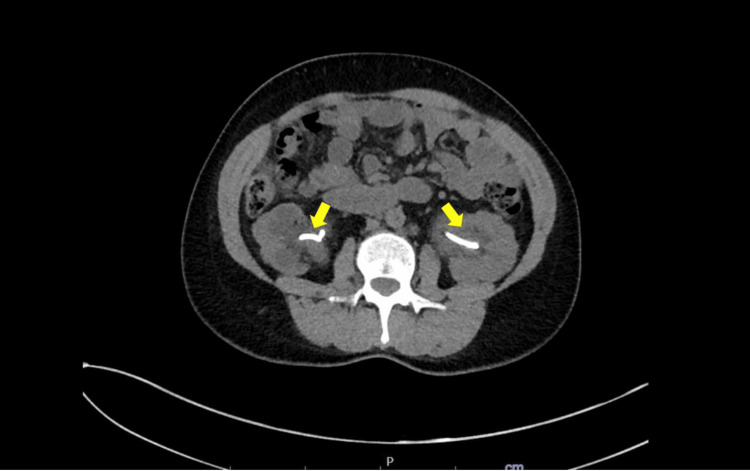
Cross-sectional view of CT abdomen and pelvis showing the resolution of obstruction after placement of ureteral stents.

Bilateral stent replacement was performed, which resulted in symptomatic relief (Figures 3, 4). Bilateral ureteral stent placement was planned and on the day of this procedure, her creatinine and eGFR levels were 2.36 mg/dL and 27.1 mL/min/1.73 m², respectively. After placement of the stents, during the time of discharge, creatinine decreased to 1.49 mg/dL and eGFR improved to 47 mL/min/1.73 m² (Table 1). The patient remains stable and drug-free; however, she continues to rely on ureteral stents to manage ongoing ureteral obstructions and hydronephrosis. Regular follow-up is required to monitor her renal function and prevent further complications related to her prior ketamine use.

## Discussion

Ketamine abuse is increasingly recognized as a significant cause of uropathy, presenting a spectrum of complications that can severely impact the urinary system. Fibrosis of the bladder and ureters is a common consequence, leading to ureteral obstruction and subsequent hydronephrosis. Studies by Chu et al. have shown that ketamine-associated uropathy affects both the lower and upper urinary tracts, with 51% of patients exhibiting unilateral or bilateral hydronephrosis as detected by ultrasonography [5]. Persistent obstruction and inflammation can escalate to renal damage, manifesting as elevated serum creatinine levels and reduced glomerular filtration rate (GFR). Inflammation can extend to the surrounding tissues, leading to periureteral and perinephric fat stranding, which indicates severe inflammation in imaging studies.

The clinical presentation often mimics more common conditions such as urinary tract infections (UTIs), with secondary infections occurring in up to 30% of cases [6]. This resemblance can delay accurate diagnosis and appropriate management, as antibiotics do not address the underlying ketamine-induced damage. Early assessment of potential ketamine abuse is crucial in patients with a history of substance abuse, as proposed by Lamers et al. [7]. Regular follow-up is advisable for patients with ketamine-associated hydronephrosis, as they are at a higher risk of developing renal function decline compared to those without hydronephrosis [8].

Management strategies for ketamine-associated uropathy focus on symptomatic relief and complication prevention. Ureteral stent placement is commonly employed to alleviate obstruction, while antibiotic therapy addresses secondary infections. Surgical interventions may be required to correct strictures or remove scar tissue. Reducing or eliminating ketamine use is essential to prevent further damage. Pharmacological treatments such as anticholinergics, beta-3 adrenoceptor agonists, and botulinum toxin A injections are used to manage urinary symptoms. Advanced cases with significant hydronephrosis and impaired renal function may necessitate ureteral stenting or percutaneous nephrostomy [9].

Regular follow-up is imperative for patients with ketamine-associated hydronephrosis to monitor renal function and prevent further decline [8]. Despite these interventions, early detection of this syndrome remains challenging, with many patients presenting to specialist centers only after significant damage has occurred, having missed the opportunity where symptoms could have resolved with abstinence alone.

## Conclusions

As drug abuse, particularly ketamine, becomes increasingly common, it is crucial to understand its multifaceted impact on health. While ketamine is known for causing significant damage to the kidneys and liver, its severe and often overlooked ureteral complications also warrant attention. The present case highlights the persistent and substantial burden of these complications, even after the cessation of ketamine use. Both patients and healthcare providers must stay alert to these issues, integrating them early into the differential diagnosis to avoid missed diagnoses and improve management outcomes. Enhanced awareness and prompt detection are key to effectively addressing and mitigating the long-term consequences of ketamine abuse.
